# Hepatitis delta testing trends in a US national cohort: An analysis of patient and provider-level predictive factors

**DOI:** 10.1097/HC9.0000000000000401

**Published:** 2024-04-12

**Authors:** Binu V. John, Mahmoud Manouchehri Amoli, Donna M. Evon, Robert Wong, Bassam Dahman

**Affiliations:** 1 Department of Medicine, Division of Gastroenterology and Hepatology, Miami VA Medical System, Miami, Florida, USA; 2 Division of Medical Education, University of Miami Miller School of Medicine, Miami, Florida, USA; 3 Department of Health Behavior and Policy, Virginia Commonwealth University, Richmond, Virginia, USA; 4 Department of Medicine, Division of Gastroenterology and Hepatology, University of North Carolina, Chapel Hill, North Carolina, USA; 5 Division of Gastroenterology and Hepatology, Palo Alto VA Health System, Alto, California, USA; 6 Division of Gastroenterology, Stanford University, Palo Alto, California, USA

## Abstract

**Background:**

The low prevalence of HDV infection in the United States could be attributed to insufficient testing rate, which can result in an underestimation of the true burden of HDV. The primary objective of this study is to quantify the prevalence of and factors associated with HDV antibody (anti-HDV) or RNA testing, among participants with positive HBsAg in the Veterans Health Administration (VHA).

**Methods:**

We conducted a retrospective cohort study of participants who tested positive for HBsAg between January 2000 and December 2022 within the VHA. We identified those who were tested for HDV, and patient and provider-level factors associated with HDV testing.

**Results:**

Of 41,658 participants with positive HBsAg who had follow-up, 4438 (10.7%) were tested at least once for HDV, of which 135 (3.0%) were positive. Participants in the Northeast (adjusted odds ratio [aOR]: 1.30, 95% CI: 1.17–1.44, *p*<0.001), and receiving hepatology care (aOR: 1.38, 95% CI: 1.24–1.54, *p*<0.001) were more likely, while those in the Midwest (aOR: 0.69, 95% CI: 0.60–0.79, *p*<0.001), under the care of a primary care provider (aOR: 0.61, 95% CI: 0.50–0.74, *p*<0.001), Blacks (aOR: 0.85, 95% CI: 0.77–0.94, *p*=0.001), participants who were HCV antibody–positive (aOR: 0.89, 95% CI: 0.81–0.99, *p*=0.03), and participants who were HIV-positive (aOR: 0.80, 95% CI: 0.71–0.90, *p*<0.001) were less likely to be tested for HDV.

**Conclusions:**

HDV screening rates in the VHA remain low overall. Participants who are Black, living in the Midwest, patients who are HIV-positive, and patients who are HCV-positive are less likely to be tested for HDV. These results suggest that risk-based screening strategies are ineffective in the VHA and highlight the need for refining testing strategies to increase HDV screening rates.

## Graphical abstract

**Figure:**
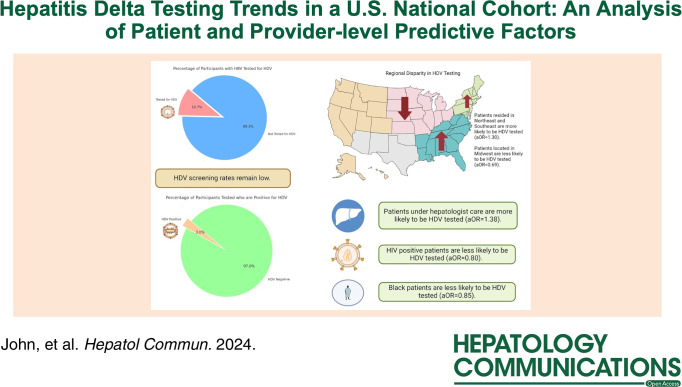

## INTRODUCTION

HDV is a coinfection that occurs in 3.8%–8.4% of patients with HBV infection, with an estimated prevalence of ~15 million people worldwide and is associated with rapid progression to cirrhosis, liver failure, and HCC within 5–10 years.^1^,^2^,^3^,^4^ With novel and well-tolerated HDV-specific treatments (bulevirtide, lonafarnib) on the horizon, early identification of HDV would have a significant impact on preventing progression to cirrhosis and HCC in infected patients.^5^,^6^

Despite the considerable burden of HDV, it remains undertested and underdiagnosed. Previous studies have revealed low rates of HDV testing among patients with chronic hepatitis B in the United States.^7^,^8^,^9^ In a study published by the Veterans Health Administration (VHA) examining HDV screening between 1999 and 2013, investigators found that 2175 (8.5%) out of 25,603 patients with a positive HBsAg were tested for HDV.^7^ However, the epidemiology of both HBV and HDV, and testing rates have changed since 2013, as younger individuals who are more likely to be vaccinated for HBV enter VHA care, and the recognition of HDV increases.

Despite low screening rates in national cohorts, the American Association for the Study of Liver Diseases (AASLD) guidelines do not recommend universal testing of all patients with HBV, because of limited data on the benefits of universal testing.^10^ The 2018 AASLD HBV Guidelines recommend testing of persons who are HBsAg‐positive at risk for HDV, including those with HIV infection, persons who inject drugs, men who have sex with men, immigrants from areas of high HDV endemicity, and patients who are HBsAg‐positive with low or undetectable HBV DNA but high alanine aminotransferase (ALT) levels. On the other hand, European Association of the Study of the Liver (EASL) guidelines now recommend universal testing of all patients with HBV.^11^

Therefore, the primary aim of this study was to examine and update the trend in HDV testing in the VHA, 2000–2022, and identify both patient and provider-level predictive factors influencing HDV testing rates. The ultimate goal was to assess if a risk-based screening strategy as currently recommended by the AASLD guidelines is being implemented in this US VHA-based national cohort, to inform decision-making for future screening recommendations.

## METHODS

### Study design

This was a retrospective cohort study using the Veterans Analysis of Liver Disease (VALID) cohort, with over 4,000,000 well-characterized Veterans with chronic liver disease from the VHA Corporate Data Warehouse (CDW) between January 2000 and December 2022. The cohort was assembled based on the identification of all Veterans with any International Classification of Diseases, Ninth and Tenth Revisions (ICD-9/10) code for chronic liver disease, regardless of the presence of liver enzyme abnormalities. We also included an additional 1.5 million individuals who had any endoscopic procedure in the VHA (including diagnostic or screening colonoscopy or upper endoscopy), to include controls without liver disease, who are engaged with VA care. Thus, this cohort included 4 million Veterans (out of ~9 million Veterans in the VHA). This resulted in the preliminary identification of over 67,000 out of the total 70,000 Veterans in the VHA with a positive HBsAg.

All research was conducted in accordance with both the Declarations of Helsinki and Istanbul, and the institutional review boards at the Miami and Richmond Veterans Affairs (VA) medical centers approved the study and waived the requirement for informed consent.

### Participant identification

The eligibility criteria included participants aged 18 years or older who had 1 positive HBsAg test during the study period, followed by at least 1 additional positive test for HBV (either a subsequent HBsAg, HBeAg, or HBV DNA). This was done to include only Veterans who had follow-up care for HBV in the VHA. We obtained baseline demographics (age, sex, race/ethnicity, marital status, geographic location, and insurance coverage), provider and facility characteristics (specialty of the treating physician and academic affiliations), and laboratory data as outlined in prior publications.^12^,^13^

We examined rates of testing among patients who are HBsAg-positive and at risk for HDV, and recommended for anti-HDV testing, including those with HIV infection, and individuals with substance use disorder. We identified immigrants from areas of high HDV endemicity by identifying their country of birth, as US-born, or foreign-born, classifying them as low and high HDV endemic countries.^14^ We predefined a laboratory profile of “at high-risk laboratory profile” for HDV, characterized by suppressed HBV DNA levels (<2000 IU/ml) and elevated ALT (≥2×upper limit of normal) as recommended by the AASLD guidelines.^10^ However, we were unable to identify men who have sex with men from this VA database. We extracted the count and dates of oral nucleos(t)ide antiviral prescriptions from outpatient pharmacy data. Patients were considered to have been administered oral nucleos(t)ides (lamivudine, telbivudine, adefovir, entecavir, or tenofovir) if they had filled at least 1 outpatient prescription.

Data on hepatic decompensation, HCC, cirrhosis, substance use disorder, and portal hypertension were obtained by employing the ICD (ICD-9 and ICD-10) as defined in previous publications.^15^,^16^ Alcohol Use Disorders Identification Test-Concise (AUDIT-C) scores closest to the baseline date, and yearly scores were obtained, and high AUDIT-C was defined as a score of ≥4 for men, and ≥3 for women at any point after HBV diagnosis. Diabetes status was defined using a combination of ICD-9 and ICD-10 and laboratory data.^16^ Patients were categorized as having received specialty care if they had a minimum of 1 consultation with a gastroenterology, infectious diseases, or hepatology provider within 2 years following the initial positive HBsAg result. Occurrences of death were determined using the National Death Index, with data up until the cutoff date of December 31, 2022.

### Outcomes

The primary outcome was the receipt of HDV testing (anti-HDV or HDV RNA) during the study period.

### Statistical analysis

Descriptive statistics were compared between the HDV-tested and untested groups, and *p* values were calculated using the Kruskal-Wallis test comparing the median of continuous variables or chi-squared tests for binary and categorical variables. In addition, we identified the annual trends and proportions of HDV testing among participants who are HBsAg-positive. We also employed a logistic regression model to identify the patient and provider-level factors associated with HDV testing. This model was adjusted for potential confounders such as age, sex, race, geographic location, comorbidities, provider specialty, and insurance type. Adjusted odds ratios (aORs) with 95% CIs were calculated to quantify the strength of the associations. All statistical analyses were performed using Stata version 17, and a *p* value < 0.05 was considered statistically significant.

## RESULTS

### Baseline characteristics of patients with a positive HBsAg

**FIGURE 1:**
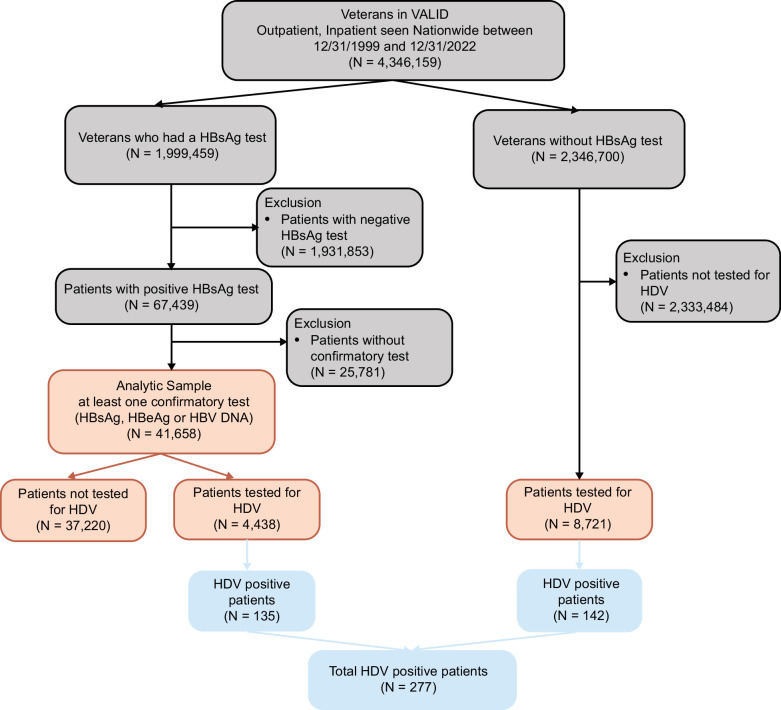
Flow diagram. Abbreviation: VALID, Veterans Analysis of Liver Disease.

A total of 67,439 participants who were HBsAg-positive were initially identified, of whom 41,658 had at least 1 repeat HBV test, and were included in the analytic sample. A total of 4438 (10.7%) of those in the analytic sample were tested for HDV, and 135 (3.0%) were anti-HDV or HDV RNA-positive (Figure 1). The majority of HDV-tested participants had anti-HDV testing, but 131 were tested for HDV RNA without anti-HDV (Supplemental Table S1, http://links.lww.com/HC9/A831). Only 28 participants who were anti-HDV–positive had HDV RNA testing, of whom 9 were HDV RNA-positive (32%) (Supplemental Table S2, http://links.lww.com/HC9/A831). In addition, 3 patients had positive HDV RNA without anti-HDV testing, and 1 patient had positive HDV PCR and negative anti-HDV. An additional 8721 participants among those without a documented hepatitis B infection within the VHA were tested for HDV, likely because they were found to have HBV on testing that occurred outside the VHA. These participants without documented HBV infection in the VHA were excluded.

For the overall cohort of participants with HBV, the median age for the total sample size was 53.9 years (Table 1). The majority of participants were US-born (99.7%), and of the 142 Veterans who were foreign-born, only 4 were from an HDV endemic country. Geographically, the largest patient population was from the Southeast (44.0%), followed by the Pacific (17.3%), South (13.2%), Northeast (13.8%), and Midwest (11.7%) regions. The majority of participants were from facilities with academic affiliations (71.9%), and were cared for by a specialist (79.2%)—primarily gastroenterology (72.1%), followed by hepatology (8.1%) and infectious diseases (7.1%); participants were being managed by a primary care provider in 12.7% of cases. With respect to their liver disease, 13.3% of patients had cirrhosis, including 6.5% with portal hypertension and 6.0% with hepatic decompensation. About 42.7% of patients had elevated ALT levels. A total of 22.7% of patients had HIV coinfection, while 47.0% had a positive HCV antibody.

**TABLE 1:** Baseline characteristics

Variable	Total (N=41,658)	HDV-tested (N=4438)	HDV not tested (N=37,220)	*p*
Demographics				
Country of birth, N (%)				
United States	41,516 (99.7)	4419 (99.6)	37,097 (99.7)	***0.389***
Low endemic (non-US)	138 (0.3)	19 (0.4)	119 (0.3)	
High endemic (non-US)	4 (0.01)	0 (0.0)	4 (0.01)	
Age at diagnosis of HBV (Median, IQR)	53.9 (14.9)	52.2 (15.9)	54.1 (14.7)	***<0.001***
Sex, male, N (%)	38,496 (92.4)	4220 (95.1)	34,276 (92.1)	***<0.001***
Race, N (%)				***<0.001***
Whites-NH	14,564 (35.0)	1400 (31.5)	13,164 (35.4)	
Blacks-NH	13,684 (32.8)	1361 (30.7)	12,323 (33.1)	
Hispanic	6597 (15.8)	622 (14.0)	5975 (16.1)	
Asians	1388 (3.3)	457 (10.3)	931 (2.5)	
Others	5425 (13.0)	598 (13.5)	4827 (13.0)	
Marital status, N (%)				***<0.001***
Married	15,826 (38.0)	1650 (37.2)	14,176 (38.1)	
Divorced/separated	15,765 (37.8)	1561 (35.2)	14,204 (38.2)	
Single	7682 (18.4)	999 (22.5)	6683 (18.0)	
Other/unknown	2385 (5.7)	228 (5.1)	2157 (5.8)	
Location, N (%)				***<0.001***
Southeast	18,335 (44.0)	1544 (34.8)	16,791 (45.1)	
Pacific	7195 (17.3)	1100 (24.8)	6095 (16.4)	
South	5500 (13.2)	438 (9.9)	5062 (13.6)	
Northeast	5737 (13.8)	1024 (23.1)	4713 (12.7)	
Midwest	4891 (11.7)	332 (7.5)	4559 (12.2)	
Insurance, N (%)				***0.001***
Yes	18,913 (45.4)	2088 (47.0)	16,825 (45.2)	
No	18,573 (44.6)	1970 (44.4)	16,603 (44.6)	
Unknown	4172 (10.0)	380 (8.6)	3792 (10.2)	
Medicaid eligibility, N (%)				***<0.001***
Yes	635 (1.5)	113 (2.5)	522 (1.4)	
No	40,139 (96.4)	4208 (94.8)	35,931 (96.5)	
Unknown	884 (2.1)	117 (2.6)	767 (2.1)	
Provider and facility characteristics				
Specialty, N (%)				***<0.001***
Primary care	5302 (12.7)	119 (2.7)	5183 (13.9)	
Specialist	36,356 (87.3)	4319 (97.3)	32,037 (86.1)	
Specialty type, N (%)				***<0.001***
Primary care	5302 (12.7)	119 (2.7)	5183 (13.9)	
Infectious diseases	2967 (7.1)	295 (6.6)	2672 (7.2)	
Gastroenterology	30,015 (72.1)	3366 (75.8)	26,649 (71.6)	
Hepatology	3374 (8.1)	658 (14.8)	2716 (7.3)	
Academic affiliations, N (%)	29,902 (71.9)	3958 (89.2)	25,944 (69.8)	***<0.001***
Patient characteristics				
Diabetes, N (%)	16,486 (39.6)	1484 (33.4)	15,002 (40.3)	***<0.001***
High AUDIT-C, N (%)	6007 (14.4)	412 (9.3)	5595 (15.0)	***<0.001***
Substance use disorder, N (%)	26,159 (62.8)	2507 (56.5)	23,652 (63.5)	***<0.001***
HIV ever positive, N (%)	9458 (22.7)	605 (13.6)	8853 (23.8)	***<0.001***
HCVAb ever tested, N (%)	41,266 (99.1)	4392 (99.0)	36,874 (99.1)	***0.018***
HCVAb ever positive, N (%)	19,573 (47.0)	1119 (25.2)	18,454 (49.6)	***<0.001***
HCV RNA ever positive, N (%)	7958 (19.1)	553 (12.5)	7405 (19.9)	***<0.001***
Liver disease				
Cirrhosis, N (%)	5547 (13.3)	780 (17.6)	4767 (12.8)	***<0.001***
Portal hypertension N (%)	2708 (6.5)	424 (9.6)	2284 (6.1)	***<0.001***
Hepatic decompensation, N (%)	2505 (6.0)	347 (7.8)	2158 (5.8)	***<0.001***
HCC, N (%)	1350 (3.2)	200 (4.5)	1150 (3.1)	***<0.001***
Hepatitis B–related factors				
Elevated ALT (≥ 2*31), N (%)	17,801 (42.7)	2303 (51.9)	15,498 (41.6)	***<0.001***
High-risk laboratory profile, N (%)	1762 (4.2)	528 (11.9)	1234 (3.3)	***<0.001***
HBV DNA ever tested, N (%)	10,744 (25.8)	3465 (78.1)	7279 (19.6)	***<0.001***
HBV DNA ever positive, N (%)	10,627 (25.5)	3461 (78.0)	7166 (19.3)	***<0.001***
Receipt of oral nucleoside/nucleotide analogs, N (%)	8247 (19.8)	2536 (57.1)	5711 (15.3)	***<0.001***
HBV DNA, N (%)				***<0.001***
>20,000 IU/mL	3835 (9.2)	1311 (29.5)	2524 (6.8)	
2000–20,000 IU/mL	1746 (4.2)	660 (14.9)	1086 (2.9)	
<2000 IU/mL	4535 (10.9)	1339 (30.2)	3196 (8.6)	
Tested negative	5981 (14.4)	723 (16.3)	5258 (14.1)	
Not tested	25,561 (61.4)	405 (9.1)	25,156 (67.6)	
HBeAg ever tested, N (%)	14,902 (35.8)	4212 (94.9)	10,690 (28.7)	***<0.001***
HBeAg ever positive, N (%)	3658 (8.8)	1260 (28.4)	2398 (6.4)	***<0.001***
HBeAb ever tested, N (%)	13,549 (32.5)	4035 (90.9)	9514 (25.6)	***<0.001***
HBeAb ever positive, N (%)	6127 (14.7)	1926 (43.4)	4201 (11.3)	***<0.001***
IgM anti-HBc ever tested, N (%)	8772 (21.1)	1245 (28.1)	7527 (20.2)	***<0.001***
IgM anti-HBc ever positive, N (%)	879 (2.1)	206 (4.6)	673 (1.8)	***<0.001***

*Note*: High AUDIT-C: AUDIT-C ≥4 for men, ≥3 for women at any time following HBV diagnosis. High-risk laboratory profile defined as elevated ALT (≥ twice upper limit of normal) and suppressed HBV DNA (<2000 IU/mL). Statistical significance (*p* value < 0.05) values are in bold/italic.

Abbreviations: ALT, alanine aminotransferase; AUDIT-C, Alcohol Use Disorders Identification Test-Concise; HCVAb, antibody to hepatitis C virus; IgM anti-HBc, IgM antibody to hepatitis B core antigen; IQR, interquantile range value comparing patients who were HDV-tested versus patients who were not HDV-tested; NH, non-Hispanic.

Notably, individuals undergoing HDV testing were relatively younger, with a median age of 52.2 years as compared to 54.1 years for those not tested for HDV (*p*<0.001); sex differences were observed, with 95.1% of HDV-tested are males compared to 92.1% males among those not tested (*p*<0.001). A notably higher percentage of Asians was noted in the HDV-tested group (10.3% vs. 2.5%, *p*<0.001), while Whites, Blacks, and Hispanics were less likely to be in the HDV-tested group. A greater proportion of single individuals were observed in the HDV-tested group (22.5% vs. 18.0%, *p*<0.001).

Regional disparities were apparent, with more participants in the HDV-tested group from the Pacific region (24.8% vs. 16.4%, *p*<0.001) and less in the Southeast (34.8% vs. 45.1%, *p*<0.001). Significant differences were observed in specialty type and academic affiliations, with HDV-tested Veterans more likely to be managed by specialists (97.3% vs. 86.1%, *p*<0.001), at centers with academic affiliations (89.2% vs. 69.8% not tested, *p*<0.001). Diabetes was less common in the HDV-tested group, (33.4% vs. 40.3%, *p*<0.001), as was alcohol misuse, defined as participants with high AUDIT-C (9.3% vs. 15.0%, *p*<0.001). On the other hand, HDV-tested individuals were more likely to have cirrhosis (17.6% vs. 12.8%, *p*<0.001) and portal hypertension (9.6% vs. 6.1%, *p*<0.001).

Elevated ALT was more common in the HDV-tested group (51.9% vs. 41.6%, *p*<0.001), as were participants with elevated ALT and suppressed HBV DNA (11.9% vs. 3.3%, *p*<0.001).

### Hepatitis D testing rates by year

**FIGURE 2:**
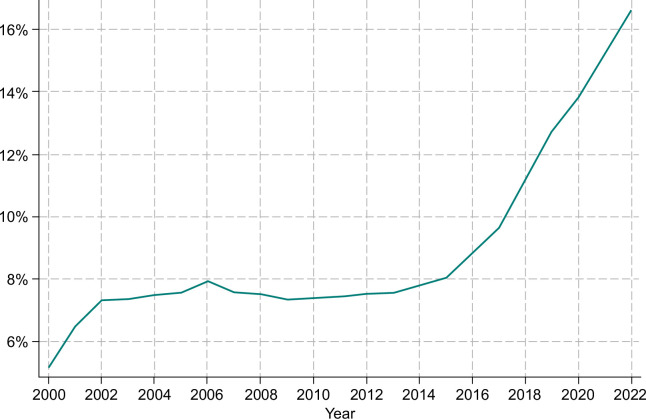
Proportion of participants who were HBV-positive and who were tested for HDV.

Figure 2 shows the proportion of all participants with HBV, who have been screened for HDV at least once. We observe that the proportion of participants with HBV who were screened for HDV increased from <6% to ~8% between 2000 and 2006, plateaued at that level from 2006 to 2015, and increased from 8% in 2015 to 16% in 2022 (Figure 2).

**FIGURE 3:**
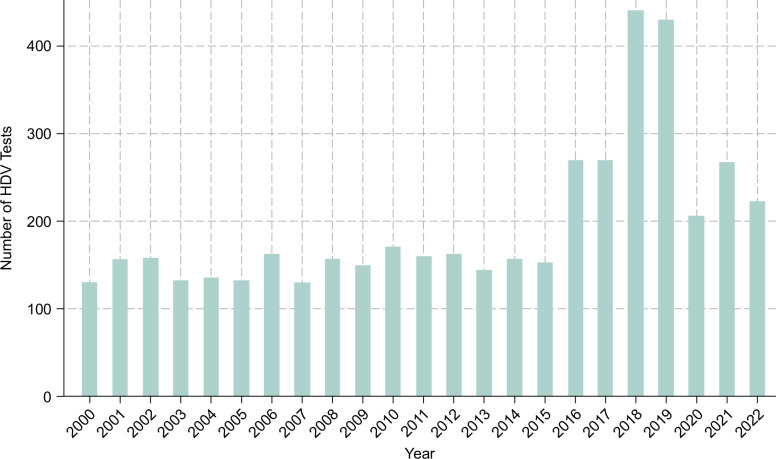
Annual number of HDV tests among participants who were HBsAg-positive.

Figure 3 shows the annual number of HDV tests performed in the VHA between 2000 and 2022. The results also show that the number of participants tested for HDV each year was stable at ~150 a year between 2000 and 2015. This increased to ~275 tests a year from 2016 to 2017, and further increased to >400 tests year from 2018 to 2019, before dropping to ~200 tests a year during the COVID-19 pandemic (2020–2022) (Figure 3).

The significant rise in HDV testing observed in 2016 was associated with an increasing engagement of patients with specialists in Gastroenterology and Hepatology. The number of patients with HBV receiving HBV care from gastroenterologists in 2016 showed a marked increase, almost doubling from the previous year. Also, after 2016, there was a sustained and significant increase in the average rate of care of patients with HBV by specialists (gastroenterologists, hepatologists, and Infectious Diseases).

### Patients and provider characteristics associated with HDV testing

**FIGURE 4:**
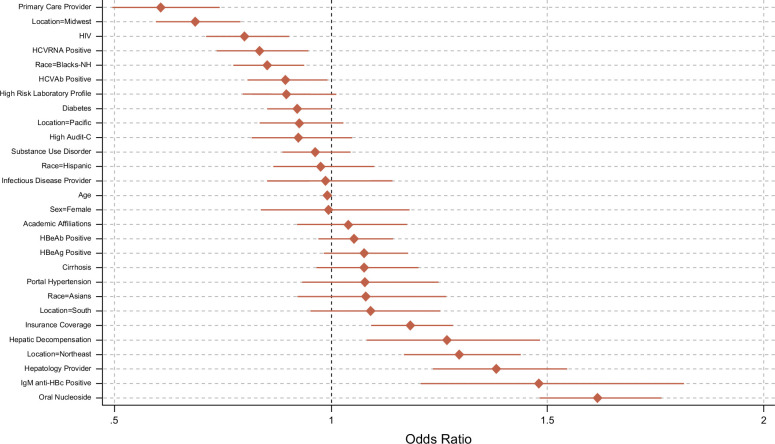
Patient and provider characteristics associated with HDV testing in participants with hepatitis B infection in the VA system. Abbreviations: AUDIT-C, Alcohol Use Disorders Identification Test-Concise; HCVAb, antibody to hepatitis C virus; IgM anti-HBc, IgM antibody to hepatitis B core antigen; NH, non-hispanic.

The logistic regression analysis results indicate that several demographic, geographical, clinical, and health care–related factors significantly influence the rate of HDV testing among participants with positive HBsAg (Table 2 and Figure 4).

**TABLE 2:** Predictors of HDV testing using logistic regression

(N=41,658)	Adjusted odds ratio	95% CI	*p*
Age at diagnosis of HBV	0.99	(0.99,0.99)	***<0.001***
Sex (ref. Male)	0.99	(0.84,1.18)	0.945
Race (ref: Whites-NH)			
Blacks-NH	0.85	(0.77,0.94)	***0.001***
Hispanic	0.98	(0.87,1.10)	0.683
Asians	1.08	(0.92,1.26)	0.339
Others	0.93	(0.83,1.05)	0.243
Marital status (ref. Married)			
Divorced/separated	1.00	(0.92,1.10)	0.922
Single	1.05	(0.95,1.17)	0.322
Other/unknown	0.95	(0.80,1.12)	0.544
Location (ref. Southeast)			
Pacific	0.93	(0.84,1.03)	0.154
South	1.09	(0.95,1.25)	0.210
Northeast	1.30	(1.17,1.44)	***<0.001***
Midwest	0.69	(0.60,0.79)	***<0.001***
Insurance			
Yes	1.18	(1.09,1.28)	***<0.001***
Unknown	0.93	(0.81,1.07)	0.300
Specialty type (ref. Gastroenterology)			
Primary care	0.61	(0.50,0.74)	***<0.001***
Infectious diseases	0.99	(0.85,1.14)	0.858
Hepatology	1.38	(1.24,1.54)	***<0.001***
Academic affiliation (ref. nonacademic)	1.04	(0.92,1.18)	0.525
Diabetes	0.92	(0.85,1.00)	***0.046***
High AUDIT-C	0.92	(0.82,1.05)	0.222
Substance use disorder	0.96	(0.89,1.05)	0.370
HIV-positive	0.80	(0.71,0.90)	***<0.001***
HCVAb ever tested	1.33	(0.95,1.88)	0.100
HCVAb ever positive	0.89	(0.81,0.99)	***0.031***
HCV RNA ever positive	0.83	(0.74,0.95)	***0.005***
Cirrhosis	1.08	(0.97,1.20)	0.185
Portal hypertension	1.08	(0.93,1.25)	0.312
Hepatic decompensation	1.27	(1.08,1.48)	***0.003***
High-risk laboratory profile	0.90	(0.80,1.01)	0.073
HBV DNA tested	0.59	(0.21,1.68)	0.321
HBV DNA positive	4.72	(1.66,13.44)	***0.004***
Receipt of oral nucleoside/nucleotide analogs	1.62	(1.48,1.76)	***<0.001***
HBeAg ever tested	7.84	(6.43,9.55)	***<0.001***
HBeAg ever positive	1.08	(0.98,1.18)	0.112
HBeAb ever tested	2.41	(2.05,2.82)	***<0.001***
HBeAb ever positive	1.05	(0.97,1.14)	0.209
IgM anti-HBc ever tested	1.49	(1.35,1.63)	***<0.001***
IgM anti-HBc ever positive	1.48	(1.21,1.82)	***<0.001***

*Note*: High-risk laboratory profile: elevated ALT (≥ twice upper limit of normal) and suppressed HBV DNA (<2000 IU/mL). Substance use disorder identified using ICD-9 and ICD-10. Bold/italic indicates *p* value < 0.05.

Abbreviations: ALT, alanine aminotransferase; AUDIT-C, Alcohol Use Disorders Identification Test-Concise; HCVAb, antibody to hepatitis C virus; IgM anti-HBc, IgM antibody to hepatitis B core antigen; NH, non-Hispanic.

Participants in the Northeast (aOR: 1.30, 95% CI: 1.17–1.44, *p*<0.001), and receiving Hepatology care (aOR: 1.38, 95% CI: 1.24–1.54, *p*<0.001) were more likely, while those in the Midwest (aOR: 0.69, 95% CI: 0.60–0.79, *p*<0.001), under the care of a primary care provider (aOR: 0.61, 95% CI: 0.50–0.74, *p*<0.001), Blacks (aOR: 0.85, 95% CI: 0.77–0.94, *p*=0.001), participants who were HCV antibody–positive (aOR: 0.89, 95% CI: 0.81–0.99, *p*=0.03), and participants who were HIV-positive (aOR 0.80, 95% CI: 0.71–0.90, *p*<0.001) were less likely to be tested for HDV. Insurance coverage was a significant predictor, with participants having insurance being more likely to receive HDV testing (aOR: 1.18, 95% CI: 1.09–1.28, *p*<0.001).

Participants with hepatic decompensation were significantly more likely to undergo HDV testing (aOR: 1.27, 95% CI: 1.08–1.48, *p*=0.003). Interestingly, ever been tested for HBeAg, HBeAb, and IgM antibody to hepatitis B core antigen were strongly positively associated with HDV testing, showing aORs of 7.84 (95% CI: 6.43–9.55, *p*<0.001), 2.41 (95% CI: 2.05–2.82, *p*<0.001), and 1.49 (95% CI: 1.35–1.63, *p*<0.001), respectively. In addition, ever testing positive for IgM antibody to hepatitis B core antigen was a significant predictor of HDV testing (aOR: 1.48, 95% CI: 1.21–1.82, *p*<0.001).

### Hepatitis D testing rates among patients recommended for risk-based screening

Regarding the risk-based screening results for HDV testing based on AASLD guidelines, we found that substance use disorder did not significantly affect the likelihood of HDV testing (aOR: 0.96, 95% CI: 0.89–1.05, *p*=0.37) (Table 2). Surprisingly, individuals who were HIV-positive were significantly less likely to have HDV testing (aOR: 0.80, 95% CI: 0.71–0.90, *p*<0.001). Similarly, individuals ever positive for HCV RNA were less likely to undergo HDV testing (aOR: 0.83, 95% CI: 0.74–0.95, *p*=0.01). Since only 4 Veterans with HBV (and none who were positive for HDV) were born in an HDV endemic country, this variable was not included in the model. High-risk laboratory profile for HDV (ALT ≥ twice the upper limit of normal and HBV DNA < 2000 IU/mL) was not associated with HDV testing (aOR: 0.90, 95% CI: 0.80–1.01, *p*=0.07).

Out of 135 patients with HDV, 32 were tested for HDV RNA, with 13 testing positive. The bivariate analysis for the predictors of HDV RNA testing indicated that patients with high-risk laboratory profiles and those tested for HBV DNA were more likely to be tested for HDV RNA (Supplemental Table S3, http://links.lww.com/HC9/A831). However, an analysis of those who were HDV RNA tested revealed no predictors of a positive HDV, likely due to low numbers (Supplemental Table S4, http://links.lww.com/HC9/A831).

**FIGURE 5:**
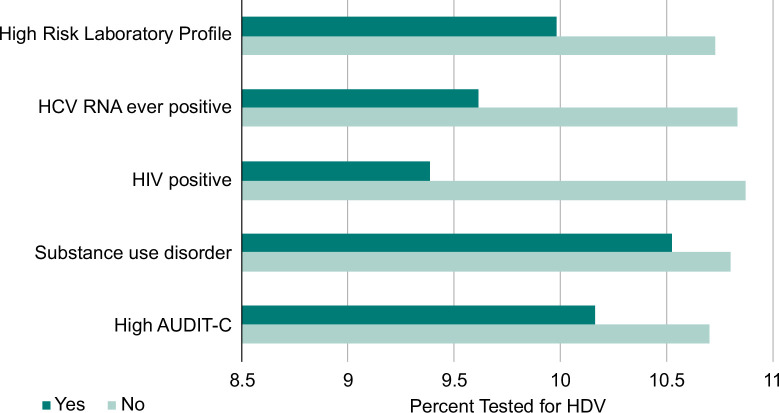
Predicted probability of HDV testing among participants eligible for risk-based screening. Abbreviation: AUDIT-C, Alcohol Use Disorders Identification Test-Concise.

The data in the predicted probability graph underscore distinct variations in HDV testing patterns relative to specific risk-based factors (Figure 5). The presence of several risk factors seemed to correlate with a diminished likelihood of patients undergoing HDV testing. For example, individuals with high-risk laboratory profiles as proposed by the AASLD guidelines for HDV had a 9.9% adjusted likelihood of being tested (compared to 10.7% in those without that profile). Similarly, individuals who were ever positive for HCV RNA had a 9.6% adjusted likelihood of testing (vs. 10.8% in those who were never positive for HCV RNA), and individuals who were HIV-positive had a 9.4% likelihood of being tested for HDV, compared to 10.9% in individuals without HIV infection. There were no significant differences in HDV testing in individuals with and without substance use disorder or moderate alcohol use.

## DISCUSSION

In this study, we examined the predictors of HDV testing among participants who were HBsAg-positive from the VA CDW. Understanding these predictors is crucial due to the association of HDV with worsened clinical outcomes and liver disease progression in HBV patients. Our study identifies key predictors of HDV testing, including race, geographical location, insurance coverage, specialty type of the provider, HIV status, and various hepatitis markers. Of note, are significant racial and geographic disparities, with Blacks and participants from the Midwest less likely to undergo HDV screening. This may reflect the relative lack of specialists caring for HBV and merit further study, particularly because there should not be significant financial barriers for Veterans regardless of race or location, to access VA services.

Our findings align with previous studies and highlight a significant concern that the existing risk-based AASLD guidelines for HDV screening among HBsAg carriers are falling short as an efficient screening strategy.^7^,^10^ The risk-based screening results show that participants with an elevated ALT with suppressed HBV DNA are not more likely to be tested, inconsistent with the guidelines. Certain factors such as being HIV-positive, and having ever tested positive for HCV RNA are linked to a reduced likelihood of testing, while substance use disorder and high AUDIT-C score did not appear to have a significant impact on HDV testing. This suggests that the use of high-risk factors to trigger a risk-based screening for HDV is not effective in this national health system, where a wide variety of providers take care of patients with HBV. Approximately 12% of Veterans with HBV are being managed by primary care, who may not be familiar with the guidelines. On the other hand, receipt of care from a hepatology specialist was associated with a higher likelihood of HDV testing, which may reflect the provider’s familiarity with HDV and guideline recommendations.

As we anticipate the introduction of new treatments for HDV infection, the evidence supports the necessity to transition toward a universal HDV screening approach for all HBsAg carriers in the United States. This shift could enhance detection rates and better align screening practices with the upcoming therapeutic advancements in the field.

Our findings show a low rate of HDV testing, emphasizing a critical gap in current practices, where many patients with HBV are not receiving the necessary screening. Our annual rates of HDV testing in the VHA indicate that rates of HDV testing were gradually increasing between 2016 and 2019, but dropped significantly between 2020 and 2022. We believe that the increased rate of HDV testing was associated with an increasing number of patients with HBV who were engaged with specialist care and that the drop between 2020 and 2022 may be secondary to the impact of the COVID-19 pandemic. Despite the excitement in the field about the impending arrival of newer antiviral medications, ~90% of Veterans with HBV have not received screening for HDV. Ina addition, we observed that the rate of HDV testing was associated with the quality of HBV care. Veterans who received a second HBV confirmatory test were more likely to be HDV-tested. The results are consistent with other findings in VA health care that suggest suboptimal adherence to guideline-recommended HBV care.^17^ In another study, Kaplan et al^18^ reported increases in guideline-recommended process measures over time, yet it also highlighted the need for improvement in HBV DNA testing and HCC surveillance among Veterans. The differences in follow-up care represent a significant concern and an opportunity for systemic improvement.

Our findings corroborate 2 prior findings of HDV screening in the VHA. In the paper by Kushner et al^7^ that examined HDV screening in the VHA before 2013, rates of overall HDV screening were 8.5%. At that point in time, only 25,603 participants with HBV were identified in the VHA. The current study includes over 41,000 participants, but the overall screening rates for HDV remain ~10%. A more recent paper by Wong et al^9^ found that among 12,002 Veterans with documented chronic HBV, 19.7% received HDV testing. Our study examines all patients with HBV who had at least 1 confirmatory test (without the need for a 6-month follow-up), which explains the higher number of participants.

We acknowledge the following limitations of our study. First, due to the retrospective study design, there is a possibility of residual confounding that may affect the results. Second, over 8000 participants without documented HBV in the VHA underwent HDV testing, of whom 142 were anti-HDV–positive. This indicates that some of the HBV and HDV testing could have been performed outside the VHA, which could not be captured in this study. Third, the VHA has a male-predominant population, and a higher prevalence of substance misuse. However, no sex-based differences in testing rates have been described, and this is unlikely to have influenced screening rates or predictors of screening. Fourth, poor-quality HBV care within the VHA is a much more serious problem than inadequate HDV testing. One should not be surprised that few patients who were HBsAg-positive were tested for HDV if they were not even tested for HBV DNA. To minimize this, we restricted our sample to Veterans who had at least 1 additional follow-up HBV testing after initial diagnosis of HBV. Finally, we acknowledge the incomplete capture of risk factors for HDV, notably of men who have sex with men. Although we were unable to capture residence in countries with a high prevalence of HDV, we were able to use country of birth as a surrogate, and found that only 4 Veterans (and none of the patients who were HDV-positive) were born in an HDV endemic country.

Our study also has relative strengths. The large sample of over 41,000 participants accrued over 22 years represents screening within a single health system and indicates trends over time, rather than a cross-sectional snapshot. Our findings that participants are at increased risk of HDV, such as those with HCV and HIV are at decreased rather than increased likelihood of screening, highlight the need for universal HDV screening of all patients with HBV.^19^,^20^ Finally, the inclusion of data from the COVID era indicates the setbacks in HDV screening that are likely attributable to the pandemic and highlights the need for greater education of providers on HDV. Our data support recommendations by several experts, highlighting the need for increased HDV testing and consideration of reflex anti-HDV testing, both in the United States and worldwide.^21^,^22^,^23^

In conclusion, HDV screening rates in the VHA remain low overall, with a drop in annual screening rates during the COVID-19 pandemic. Participants who are Black, living in the Midwest, patients who are HIV-positive, and patients who are HCV-positive are less likely to be tested for HDV. These results suggest that risk-based screening strategies are ineffective in the VHA and highlight the need to consider universal testing strategies to increase HDV screening rates.

## Supplementary Material

## Data Availability

The United States Department of Veterans Affairs (VA) places legal restrictions on access to Veterans’ health care data, which includes both identifying data and sensitive patient information. The analytic data sets used for this study were not permitted to leave the VA firewall without a Data Use Agreement. This limitation is consistent with those of other studies based on VA data. However, VA data are freely available to researchers behind the VA firewall, with an approved VA study protocol. For more information, please visit https://www.virec.research.va.gov or contact the VA Information Resource Center (VIReC) at Virec@Va.gov. The data included here have not been published before. An abstract containing these data has been selected for presentation as a poster of distinction at the American Association of Study of Liver Diseases meeting at Boston in November 2023. Binu V. John and Bassam Dahman had full access to all the data in the study and were responsible for the integrity of the data and the accuracy of the data analysis. Concept and design: Binu V. John. Acquisition, analysis, and interpretation of data: Binu V. John, Mahmoud Manouchehri Amoli, and Bassam Dahman. Drafting of the manuscript: Binu V. John and Mahmoud Manouchehri Amoli. Critical revision of the manuscript for important intellectual content: All authors. Statistical analysis: Mahmoud Manouchehri Amoli and Bassam Dahman. Obtained funding: Binu V. John. Administrative, technical, or material support: All authors. Supervision: Binu V. John and Bassam Dahman. Services supporting this analysis and interpretation of data of this research project were supported through an investigator-initiated research study grant by Gilead. Services supporting this analysis and interpretation of data of this research project were also generated by the VCU Massey Cancer Center Biostatistics Shared Resource, supported in part by funding from the NIH-NCI Cancer Center Support Grant P30 CA016059. Binu V. John received institutional research support from Exact Sciences, Gilead, and Glycotest, Inc. Robert Wong received institutional research support from Gilead Sciences, Exact Sciences, Thera Technologies, and Durect Corporation. The remaining authors have no conflicts to report.
