# Supplementary material for: Hepatitis delta testing trends in a US national cohort: An analysis of patient and provider-level predictive factors
Source: Hepatol Commun. 2024 Apr 12;8(5):e0401. doi: 10.1097/HC9.0000000000000401 (PMC11019823; doi:10.1097/HC9.0000000000000401)
Supplement: Supplementary file 1 [file hc9-8-e0401-s001.docx]

Supplementary Table 1: Distribution of number of patients tested for HDV RNA and anti-HDV among 4,438 patients tested for HDV

|  | | HDV RNA | | |
| --- | --- | --- | --- | --- |
|  |  | Tested | Not Tested | Total |
| Anti-HDV | Tested | 291 | 4,016 | 4,307 |
|  | Not Tested | 131 | 37,220 | 37,351 |
|  | Total | 422 | 41,236 | 41,658 |

Supplementary Table 2: Distribution of test result status for HDV RNA and anti-HDV among 4,438 Patients Tested for HDV

|  | | HDV RNA | | | |
| --- | --- | --- | --- | --- | --- |
|  |  | Positive | Negative | Not Tested | Total |
| Anti-HDV | Positive | 9 | 19 | 122 | 150 |
|  | Negative | 1 | 262 | 3,894 | 4,157 |
|  | Not Tested | 3 | 128 | 37,220 | 37,351 |
|  | Total | 13 | 409 | 41,236 | 41,658 |

Supplementary Table 3: Predictor of HDV RNA testing among HDV positive

|  | Bivariate Model | |
| --- | --- | --- |
| (N=135) | Adjusted OR (95% CI) | P Value |
| Age at diagnosis of HBV | 0.98 (0.95,1.02) | 0.309 |
| Sex (ref. Male) | NA | NA |
| Race (ref: Whites-NH) |  |  |
| Blacks-NH | 1.29 (0.48,3.45) | 0.617 |
| Hispanic | 1.23 (0.36,4.21) | 0.736 |
| Asians | 0.63 (0.07,5.83) | 0.688 |
| Others | 1.33 (0.42,4.27) | 0.628 |
| Marital status (ref. Married) |  |  |
| Divorced/Separated | 0.74 (0.29,1.90) | 0.529 |
| Single | 0.76 (0.28,2.09) | 0.601 |
| Other/Unknown | 1.08 (0.19,6.09) | 0.928 |
| Location (ref. Southeast) |  |  |
| Pacific | 2.30 (0.78,6.79) | 0.131 |
| South | 1.62 (0.27,9.75) | 0.599 |
| Northeast | 1.05 (0.33,3.33) | 0.928 |
| Midwest | 2.43 (0.49,12.11) | 0.279 |
| Insurance |  |  |
| Yes | 1.51 (0.66,3.47) | 0.330 |
| Unknown | 4.00 (0.96,16.66) | 0.057 |
| Specialty type (ref. Gastroenterology) |  |  |
| Primary care | NA | NA |
| Infectious Diseases | 0.40 (0.05,3.30) | 0.398 |
| Hepatology | 2.42 (0.80,7.30) | 0.116 |
| Academic Affiliation (ref. non-academic) | 3.04 (0.38,24.50) | 0.295 |
| Diabetes | 0.63 (0.24,1.67) | 0.351 |
| High AUDIT-C | 1.49 (0.37,5.96) | 0.575 |
| Substance abuse disorder | 0.71 (0.30,1.67) | 0.431 |
| HIV positive | 0.17 (0.02,1.29) | 0.086 |
| Anti-HCV ever tested | NA | NA |
| Anti-HCV ever positive | 0.86 (0.39,1.94) | 0.723 |
| HCV RNA ever positive | 0.91 (0.37,2.22) | 0.834 |
| Cirrhosis | 2.13 (0.96,4.72) | 0.062 |
| Portal hypertension | 1.70 (0.70,4.16) | 0.244 |
| Hepatic decompensation | 0.69 (0.22,2.19) | 0.533 |
| High risk laboratory profile | 2.86 (1.27,6.43) | *0.011* |
| HBV DNA tested | 5.01 (1.44,17.42) | *0.011* |
| HBV DNA Positive | NA | NA |
| Receipt of oral nucleoside/nucleotide analogues | 2.00 (0.86,4.67) | 0.110 |
| HBeAg ever tested | NA | NA |
| HBeAg ever positive | 0.48 (0.17,1.36) | 0.169 |
| HBeAb ever tested | NA | NA |
| HBeAb ever positive | 0.80 (0.37,1.75) | 0.582 |
| HBcIgM ever tested | 2.04 (0.89,4.67) | 0.093 |
| HBcIgM ever positive | NA | NA |

Note: High risk laboratory profile: Elevated ALT (>=2*31 upper limits) and suppressed HBV DNA (<2000 IU/ML)

Substance abuse disorder identified using ICD-9 and ICD-10. *Italic* indicates p-value <0.001

Supplementary Table 4: Predictors of HDV RNA Positive Status among HDV RNA

Tested

|  | Bivariate Model | |
| --- | --- | --- |
| Parameters | Adjusted OR (95% CI) | P Value |
| Number of Patients | 32 |  |
| HDV Positive | 13 |  |
| Age at First HDV Testing | 1.02 (0.95,1.09) | 0.577 |
| Sex (Ref: Female) | NA | NA |
| Race / Ethnicity (Ref: White) |  |  |
| Black | 2.00 (0.27,14.70) | 0.496 |
| Hispanic | 5.25 (0.49,56.80) | 0.172 |
| Other | 4.67 (0.53,40.89) | 0.164 |
| Diabetes (Ref: No) |  |  |
| Yes | 1.60 (0.27,9.53) | 0.606 |
| Substance Use Disorder (Ref: No) |  |  |
| Yes | 0.57(0.13,2.60) | 0.469 |
| AUDIT-C Score (Ref: Low) |  |  |
| High | 3.27 (0.26,40.47) | 0.355 |
| HIV Co-infection (Ref: No) | NA |  |
| HCV Ab Positive (Ref: No) |  |  |
| Yes | 0.93 (0.22,4.00) | 0.926 |
| High Risk Individual * (Ref: No) |  |  |
| Yes | 0.95 (0.23,3.92) | 0.946 |
| Elevated ALT (Ref: No) |  |  |
| Yes | 1.19 (0.23,6.17) | 0.835 |

Abbreviations: AUDIT-C - Alcohol Use Disorders Identification Test-Concise; aOR – adjusted Odd Ratio; CI: Confidence interval.

Note: High risk of HDV defined as ALT>=2*31 Upper limits and HBV DNA <2000 IU/ML. Other race included, Native American / Alaska Native, Native Hawaiian, Pacific Islander, and two or more races.

Statistical method/test applied: Logistic model. Values in bold denote statistical significance.

NA cannot be assed due to limited sample size in the bivariate analysis.
